# The New York Daily Times and the Dissection Bill

**Published:** 1854-03

**Authors:** 


*The New York Daily Times and the Dissection Bill.*—We couple these two names together, because the course of that paper has entitled it to a favorable mention from the medical journalist, and, moreover, because when the dissection bill passes, as we hope and believe it will, the New York Daily Times will be entitled to a large modicum of credit for its services to the cause of science.

Ever since this bill came before the Senate, the Times has consistently, boldly, and forcibly advocated before the people and the legislature, the necessity and justice of its passage. It took the ground when it was unpopular. It did not wait for the public sentiment to declare itself, but it turned to with a will to mould and form that sentiment. It did not hesitate to state the thing fairly and without abatement. It took the open ground, asserting that the bill was a benefit, not to the profession, but to the people: that the sanctity of the grave, and the abolition of the trade of the resurrectionist, depended upon its success.

We cannot help mentally contrasting this open and manly course of a secular paper, with the miserably mean attempt of a medical periodical in the same city, to depreciate the bill by calling it a college monopoly, and throwing out the hideous insinuation that medical schools will make a speculation of the trade in dead bodies, by selling them to the profession at high prices. Such an idea never came from an honorable man. It would never have suggested itself as possible to a mind of any sense of refinement or honor, and is but the pitiable evidence of a heart full of envy, malice, and all uncharitableness.

To recur to the pleasanter subject upon which we started. The N. Y. Daily Times has a medical department, conducted with considerable vigor, which is a common deposit for the floating medical news of the day. This evidence of a wish to adapt the Times to the wants of physicians, will, we hope, find its reward in a cordial support from the profession.

But the Times is not alone in this matter. Very many other of the daily papers have shown a proper appreciation of the rights of the profession, by giving their support to the dissection bill. Among these the Commercial Advertiser, of this city, has had numerous well-written and convincing editorial articles on the bill.

We mention these things with pleasure. The better and more independent portion of the secular press have shown a good spirit, at the expense of some obloquy and unpopularity. But a manly and plain-spoken press will always vindicate itself; and we have written these few sentences of just praise, only as an indication of our appreciation of the services rendered by these journalists. H.

